# Assessing Field Hockey Face Mask Performance

**DOI:** 10.1007/s10439-025-03794-z

**Published:** 2025-07-11

**Authors:** Natasha Bialecki, Rory England, Jon Farmer, Sean Mitchell, Ellie Rayer, Paul Sherratt

**Affiliations:** Wolfson School of Mechanical, Electrical and Manufacturing Engineering, Loughborough University, Loughborough, United Kingdom

**Keywords:** Field hockey, Face mask, Penalty corner, Head injury, Impact testing

## Abstract

**Purpose:**

The purpose of this study was to investigate the protective performance differences of commercially available field hockey face masks whilst proposing and evaluating a methodology using commonly available equipment suitable for use as a dynamic performance test standard.

**Methods:**

Field hockey balls were propelled at realistic and repeatable speeds (26.8 ± 0.25 ms^-1^ and 35.8 ± 0.25 ms^-1^; mean ± SD), with acceptable impact location precision (± 11.3 mm; radial SD), at outfield face masks preconditioned to a range of field hockey relevant end-use temperatures. Masks were mounted on the ubiquitous Hybrid III headform and neck assembly to represent, with reasonable biofidelity, some of the potential consequences for human users at the tested impact locations.

**Results:**

Qualitative and quantitative laboratory-based measures showed that field hockey mask performance varies with speed, temperature, and impact location. Testing showed that some commercially available masks perform better than others, with key weaknesses including mechanical failure and facial contact.

**Conclusions:**

The potential for some masks to fail catastrophically, and others to provide a wide range of protection, has been demonstrated and reported to governing bodies and industry. Limitations of the equipment and methods utilised have been identified as the impetus for further work. Nonetheless, the current approach provides a testing methodology for the field hockey PPE industry.

## Introduction

Field hockey is one of the most popular team sports globally, behind only soccer [1], with an estimated 29.8 million players across five continents [2]. It is played on a rectangular pitch, usually synthetic, with a hard ball. Each team consists of 11 players (1 goalkeeper and 10 outfield players) who aim to score more goals than the opposition by using a field hockey stick to move the ball around the pitch. Players are permitted to flick, hit, push, or scoop the ball, where each method is defined in the rules of field hockey [3]. Unlike many other sports, field hockey participation is almost evenly split between genders with 52% of players being male [2].

Overall injury rates in field hockey are amongst the highest of all team sports [4–6]. Studies have found that injuries to the head, face, and neck account for 14 to 40% of field hockey injuries, varying by gender, playing level and age-group [7–14]. The risks associated with such injuries depend on factors including the specific region of the head impacted.

To reduce these risks, field hockey goalkeepers are permitted to wear helmets and outfield players may wear face masks when defending a penalty corner (PC). The use of such face masks in FIH competitions was made mandatory in 2025 [15]. The prior lack of mandate could be due to historical gameplay traditions, perceptions of risk, and not wanting to introduce new risk (e.g., unintended consequences for other players). Additionally, the paucity of extensive research into head and facial injury risk during the penalty corner means that much of the risk perception has been based on player’s anecdotal experiences. To date, only one such study has been conducted, which found that 22.4% of penalty corner related injuries are to the head [16]. Despite the lack of information on situation-specific occurrences, there is evidence that 69% of injuries above the neck occur inside the scoring ‘circle’ or within the ‘25-yard line’ (regulated regions marked on the pitch) [12].

PCs are awarded for deliberate infringements by defending players within 23 m (25 yds) of the backline; they are an important part of the game due to their scoring potential [17], despite their relatively low frequency (2.97 penalty corners per game) [17]. To overcome the PC rule limiting the first hit at goal to below the backboard height of 46 cm (~18 inches) [3], attacking players employ techniques such as the drag flick, one of the fastest [18–22] and most effective ways of scoring during a PC [23]. Thus, the current rules of field hockey are such that during a PC up to 4 outfield defending players, in addition to their goalkeeper, may rush from within the 3.88 m (12 ft) wide, 2.14 m (7 ft) high rectangular goal towards an attacking player as the attacker drag-flicks the 156–163 g, 71.3–74.8 mm diameter [24] hard field hockey ball at ~ 36.1–38.9 ms^-1^ (80–87 mph) [18–22] into the defended goal from less than 14.63 m (48 ft) away; this allows the defenders an evasive reaction time of ~ 0.38–0.41 s (less than in sports such as cricket, where the ball takes approximately 0.60 s to reach the batter from a fast bowl [25]). The reaction time may be much less if there is a deflection. The size, mass, hardness, potential proximity and elevation of the ball at these speeds poses a significant risk of head injury during a PC and justifies the use of adequate personal protective equipment (PPE), such as face masks, by the defending players.

To ensure headgear provides adequate protection, various standards organisations exist, e.g., the National Operating Committee on Standards for Athletic Equipment (NOCSAE), American Standards for Testing and Materials (ASTM), the European Committee for Standardisation (CEN) and the British Standards Institution (BSI). Whilst the organisation responsible for test standards differs by region, the methods and intended performance outcomes are largely the same. Regarding field hockey headgear, the only standard that currently exists is the NOCSAE standard for goalkeeper headgear [26]. Currently, there is no test standard for field hockey outfield face masks under any regulatory body, resulting in ambiguity about required performance levels and methods of testing. Nor is there any published research on field hockey face mask performance.

The aim of this research was to develop quantitative and qualitative test methods, informed by other sports’ standards and research testing, to assess a range of commercially available field hockey face masks to benchmark performance. Additionally, the study sought to highlight safety concerns and identify the need and/or scope for developing better field hockey-specific test methods to ensure adequate safety levels within the sport.

## Materials and Methods

### Selection of Face Mask Samples

**Fig 1:**
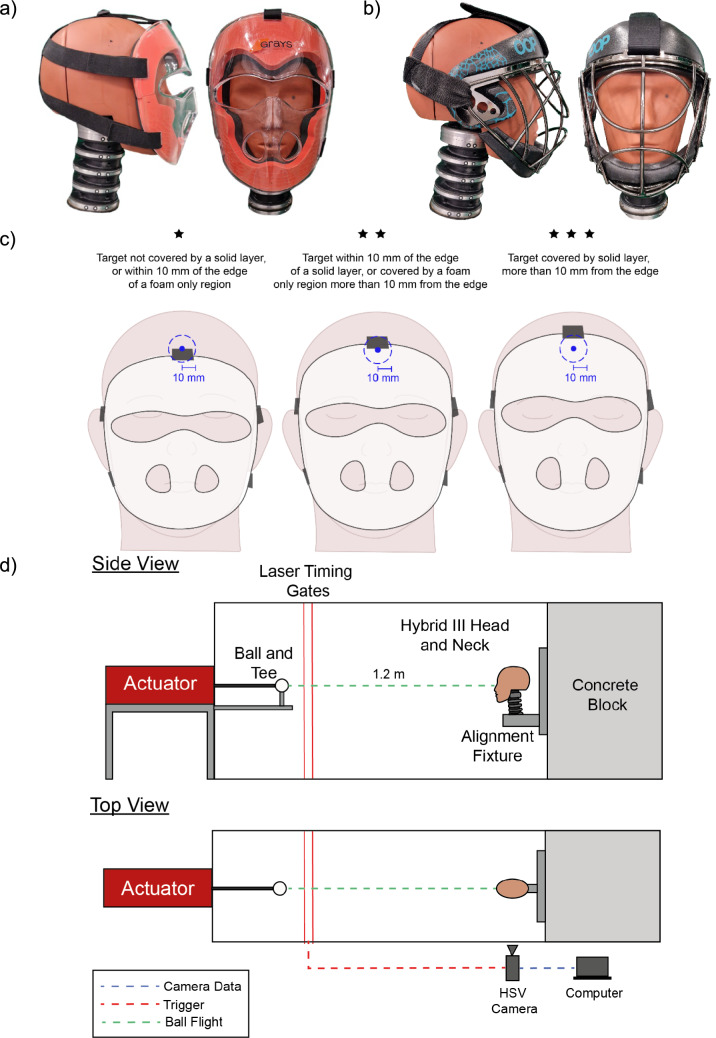
**a** Frontal and lateral view of an exemplar plastic field hockey face mask. **b** Frontal and lateral view of an exemplar metal grille field hockey face mask. **c** Coverage assessment illustration. **d** Experimental set up using custom iBPR rig

In consultation with the FIH (Fédération Internationale de Hockey (International Hockey Federation)), twelve field hockey face masks from seven brands were selected as a representative range of available masks at different price points based on brand, material, detail, price and/or willingness to provide masks for testing. Exemplar images of each of the two main styles of field hockey mask are given in Fig 1a (plastic shell construction) and Fig 1b (metal grille). To reflect the proportion of mask types (either plastic shell construction or metal grille) on the market, eleven plastic shell designs, and one metal grille design were tested.

All masks tested comprised at least one solid and one elastomeric foam impact distributing component. Mask mass, solid layer and foam layer thicknesses were measured. Mass was measured to the nearest 10 g using calibrated scales. Solid layer thickness was measured using micrometres (Linear Isleworth, England; ± 0.01 mm) in minimally curved regions and reported to ± 0.1 mm. Foam thickness was measured with vernier callipers (Linear Isleworth, England; ± 0.1 mm). Comfort liners were deconstructed to measure functional layers if they were too soft or thin (< 3 mm) to measure accurately. Foam layers were characterised by Shore O hardness (± 1). Characterisation details are provided in Table 1.

**Table 1:** Characterisation of face masks used in testing

Brand/Product	Hard Layer		Functional Soft Layer^a^					Mass (g)	Cost (£)	Certification Status
	Material	Thickness (mm)	Foam Material^a^	Thickness (mm)		Shore O Hardness				
				First Layer^f^	S Layer	First Layer^f^	S Layer			
1A	Polymer^u^	4.2	Foam^u^		12		20	410	65	✖
1B	Polymer^u^	4.5	DD^u^	10	10	51	10	310	65^p^	✖
1C	Polymer^u^	4.5	DD^u^	9	8	52	10	190	55	✖
1D	Polymer^u^	4.2	MD^u cl^		4		30	260	45	✖
2A	Polymer^u^	4.1	HD^u^		13		39	180	35	✖
2B	Polymer^u^	3.8	Foam^u^		10		45	240	50	✖
2C	Polymer^u^	3.7	Foam^u cl^		14		45	290	50^p^	✖
3A	PC	4.5	DD^u^	10	10	45	13	340	60	✔^s^
4A	Polymer^u^	4.3	DD^u^	7	7	35	33	230	50	✖
5A	Polymer^u^	3.6	Foam^u cl^		7		34	230	45	✖
6A	PC	4.7	DD^u^		17		38	290	50	✖
7A	Steel	4.5 ⌀	CC^u^		18^ m^		66	560	70	✔^s^

*PC* Polycarbonate

^a^Manufacturer description of functional energy absorbing materials (excluding very low-density comfort liners that compress > 75% with minimal force) [Key—Dual Density (DD), High Density (HD), Medium Density (MD) & Close Cell (CC)].

^cl^Comfort layer used in construction.

^u^Unspecified Polymer.

^p^Presumed price point informed from subsequent product launch.

^s^Since there is no PPE specific test standard, certification is only against a self-specified test. These were not openly shared.

^f^First layer denoted as functional layer closest to hard shell of the mask, furthest from the wearers skin.

^m^Mask used a twin shot moulding technique; therefore, it was not possible to distinguish layer thicknesses.

^⌀^Diameter of metal wire.

### Qualitative Testing

#### Fit

Prior to quantitative impact testing whilst mounted on a suitably instrumented headform and neck assembly within an instrumented ball propulsion rig (iBPR) (cf. Quantitative (Impact) Testing), masks were fitted to the headform following manufacturer’s instructions to ensure consistency with human fitting recommendations. For some masks with lower sitting constraint strap configurations, adjustments were necessary due to the non-biofidelic geometry of the selected headform in the basilar region. This adjusted fit was kept consistent throughout testing.

Fit was reviewed before impact testing to ensure no masks were disadvantaged by poor fit with the headform. Light manual manipulation (fingertip pressure/friction, primarily revealing a loose fit due to slack or slip) was applied in three directions (up-down, left-right, and twist) and fit scores awarded as follows: ⋆ very loose (> 5 mm), ⋆⋆ moderate movement under light manual manipulation in direction stated (> 2 mm), ⋆⋆⋆ no movement under light manual manipulation in direction stated (< 2 mm). Each mask was tested four times, using two examples of each model. Three researchers independently evaluated the masks according to the agreed protocol and criteria; 100% repeatability of assessment was achieved.

#### Coverage

A targeting laser mounted on the iBPR, was used to position the unmasked headform to ensure the launched ball trajectory would strike the anatomical target location. After fitting the mask to the aligned headform coverage was assessed within a 10 mm radius around the projected laser point, imitating the cricket helmet standard [27], an illustrated example is presented in Fig 1c. Holes in the mask, such as eye openings, were considered covered if the ball could not be manually passed through the aperture. Coverage scores were assigned as follows: ⋆ target not covered by a solid layer or within 10 mm of the edge of a foam only region, ⋆⋆ target covered by a foam only region more than 10 mm from the edge or within 10 mm of the edge of a solid layer, ⋆⋆⋆ target covered by a solid layer more than 10 mm from the edge (cf. Fig 1c).

#### Headform Contact and Mechanical Failure

High speed video (HSV) of subsequent ball/mask/headform impacts was used to make a qualitative visual assessment of likely direct contact between the ball and headform (direct contact) or forced contact between the mask and headform caused by the ball (mask-headform contact); where scores were assigned as follows: ⋆ ball made direct contact with the headform, ⋆⋆ ball forced the hard layer of the mask to contact the headform, or ball impacted a foam only region, ⋆⋆⋆ no clear contact. Each mask was inspected for mechanical failure after each impact and received one of the following score: ⋆ severe fracture, ⋆⋆ minor damage, ⋆⋆⋆ no visible damage.

### Quantitative (Impact) Testing

#### Equipment

A 50^th^ percentile male Hybrid III (HIII) headform, instrumented with a 6DX Pro [± 2000 g, ± 18000 deg/s] (DTS, Calabasas USA) accelerometer and gyroscope combination, was used along with the HIII neck. Data were sampled at 50 kHz, with a built-in anti-aliasing filter (− 3 dB at 10 kHz) applied. Projectiles were launched using a custom-built push-rod ball launcher (iBPR) with measured standard deviations for impact velocity and location (radially) of ± 0.25 ms^-1^ and ± 11.3 mm, respectively. The bespoke iBPR has previously been evaluated for repeatability and precision in other research [28–30], where details of its design and configuration are also documented.

The HIII neck was configured so that the Frankfort plane of the headform was horizontal (i.e., in the neutral position) with the base fixed to an adjustable alignment fixture (Fig 1d). The headform was positioned 1.2 m from the ball on the tee, aligned so that its vertical axis was normal to the intended horizontal flight path of the ball. Due to the short distance and high speeds, the ball’s trajectory has a very small 0.50–0.94° downward deviation from horizontal at impact due to gravity. The slight 5–10 mm vertical deviation was accounted for when positioning the headform.

Kookaburra Dimple Elite balls were used as projectiles and tested to confirm compliance with field hockey ball standards [24]. Each ball was used a maximum of six times in impact testing and ball velocity was measured using laser timing gates. A high-speed video (HSV) camera was positioned perpendicular to the ball flight path (Fig 1d), and captured at 15 kHz (Photron, Tokyo JP). A CFC 1000 filter was applied to linear data per SAE J211-1 [31]. The same filter was also applied to the angular velocity data, as a CFC 180 filter overly blunted the trace. This application of a CFC 1000 filter to angular data has been utilised in research on projectile head impacts in cricket [28]. Angular velocity was then differentiated, using the gradient MATLAB function, to obtain acceleration.

#### Injury Metrics and Thresholds

A range of injury metrics have been proposed in the literature, to aid the evaluation of injury risk resulting from linear motion, rotational motion, or a combination of the two. The code used for data analysis calculated 15 injury metrics, including the Gadd Severity Index (GSI) [32], Rotational Injury Criterion (RIC) [33], Power Rotational Head Injury Criterion (PRHIC) [34], Kleiven’s Linear Combination (KLC) [35], and the Generalised Acceleration Model for Brain Injury Threshold (GAMBIT) [36].

Four of the calculated injury metrics were chosen for use in this study: Resultant peak linear acceleration (PLA) [37], Head Injury Criterion (HIC15) [38], resultant peak angular acceleration (PAA) [37] and Brain Injury Criterion (BrIC) [39]. PLA and PAA were chosen for use as raw metrics, and both HIC and BrIC were chosen as some of the more widely understood composite metrics, despite their limitations in this context.

The HIC metric takes impact duration into consideration, which is not the case for PLA. It is a method of identifying the most damaging part of the acceleration pulse by finding the maximum value of the function [40] and was developed for automotive crashes, which typically involve longer duration impacts than those experienced in field hockey. BrIC examines angular velocity and includes consideration of anatomical rotation axis dependency [39]. This metric was developed for use in sports contexts (e.g., American Football), and can be utilised for field hockey, but not necessarily optimally due to the difference in impact scenarios. In the absence of research to show a proven and optimal solution for field hockey, those metrics chosen for use in this study provide exemplar published injury thresholds to compare the performance of field hockey masks, until such time as more suitable metrics and thresholds are established for the field hockey context.

For each metric, Table 2 presents threshold values associated with various injury types and risk levels, sourced directly from the literature or inferred from the published risk curves indicated, for context only, in the absence of research correlating injury and metric threshold when using a field hockey mask.

**Table 2:** Values of Peak Linear Acceleration (PLA), Head Injury Criterion (HIC15), Peak Angular Acceleration (PAA) and Brain Injury Criterion (BrIC) by types of injury and levels of injury risk

Corresponding Threshold Cited	PLA (g)	HIC	PAA (rad/s^2^)	BrIC	
				HIII	College Football Players
25% risk of skull fracture	225 [41]	1,150 [41]			
40% risk of skull fracture	**250** [41]	1,350 [41]			
5% risk of AIS 4 + injury		**700** [41]		0.76 [39]	0.69 [39]
50% risk of AIS 2 + injury	116 [42]	825 [42]	11,368 [42]	0.85 [39]	0.78 [39]
50% risk of AIS 3 + injury	162 [42]	1,442 [42]	18,775 [42]	1.02 [39]	1.02 [39]
30% risk of AIS 4 + injury		1,200 [41]		**1.00** [39]	1.02 [39]
50% risk of AIS 4 + injury		1,400 [41]		1.13 [39]	1.21 [39]
10% risk of mTBI	165 [43]	400 [43]	**9,000** [43]	0.72 [39]	0.55 [39]
25% risk of mTBI	57 [44] 200 [43]	136 [44] 498 [43]	11,000 [43]	0.78 [39]	0.65 [39]
50% risk of mTBI	79 [44] 235 [43]	235 [44] 613 [43]	5,900 [45] 13,000 [43]	0.85 [39]	0.78 [39]
75% risk of mTBI	98 [44]	333 [44] 729 [43]		0.92 [39]	0.89 [39]

Values highlighted in bold are those chosen for use in this study

The thresholds listed vary depending on their intended application (e.g., the automotive industry or sports like American football) and the methods used to calculate them. For example, the values for different levels of mTBI risk reported by King et al. (2003) [44] and Funk et al. (2007) [43] differ due to the underlying data sets used in their respective analyses.

In this study, the applicability of the tabulated thresholds to field hockey is not examined. Although Table 2 summarises a wider context for considering the results of this study, a single threshold for each metric is later cited (highlighted in bold in Table 2) for brevity and where the value has some accepted or suggested use in other contexts. For example; a PLA value of 250 g is used in British Standards for cycling and cricket [27, 46]; a HIC value of 700 is used in the US airbag standard [47]; although PAA and BrIC are not currently widely included in test standards, proposed values of 9,000 rad/s^2^ and 1.00, respectively, have been suggested in the literature [39, 43]. The use of a CFC 1000 filter on angular velocity data, rather than the SAE J211-1 [31] recommended CFC 180 filter, resulted in BrIC scores that were on average 3.9% higher. Given this small difference, it was determined that the less aggressive filter had a minimal impact on BrIC calculations and resulted in a more conservative approach to risk mitigation if compared with published thresholds that used the CFC 180 filter.

Additionally, bare headform impacts were conducted to compare masked and unmasked impact severity as suggested by each metric. This comparison adds further context meaningful to field hockey participants, i.e., how much better or worse off is a player wearing a given mask vs. not wearing a mask.

To assess the influence of face mask characteristics (Table 1) on performance, Quantitative Net Performance Scores (QuantNPS) were calculated using data from ambient temperature impacts at 60 mph across all four impact locations (the only condition in which all 12 masks were tested). For each mask, average values were calculated for each injury metric (PLA, HIC, PAA, and BrIC) across all impact locations and have been converted to a z-score, to indicate the unusualness of each mask’s performance in this cohort.

Equation 1: z-score calculation

$$z_{X,i} = \frac{{S_{X,i} - \mu_{X} }}{{\sigma_{X} }}$$

where ${S}_{X,i}$ is the raw score for metric X (PLA, HIC, PAA, or BrIC) for mask design $i$, and ${\mu }_{X}$, ${\sigma }_{X}$ are the mean and standard deviation for metric X, respectively.

Scores were then scaled from 0–100.

Equation 2: Scores scaled from 0-100

$$P_{X,i} = \left( {\frac{{\max \left( {z_{X} } \right) - z_{X,i} }}{{\max \left( {z_{X} } \right) - \min \left( {z_{X} } \right)}}} \right) \times 100$$

The worst performing mask received a score of 0, and the best performing mask received 100. All others were assigned a score proportional to their relative position between these extremes. The QuantNPS for each mask was calculated as the average of the four scaled injury metric scores, with equal weighting:

*Equation 3* *: Quantitative Net Performance Score (QuantNPS)*

$$QuantNPS_{i} = \frac{1}{4}\left( {P_{PLA,i} + P_{HIC,i} + P_{PAA,i} + P_{BrIC,i} } \right)$$

This same calculation was performed using the scores from qualitative assessments (fit, coverage, contact, and mechanical failure) to obtain Qualitative Net Performance Scores (QualNPS):

*Equation 4* *: Qualitative Net Performance Score (QualNPS)*

$$QualNPS_{i} = \frac{1}{4}\left( {P_{Fit,i} + P_{Coverage,i} + P_{Contact,i} + P_{MechFail,i} } \right)$$

QuantNPS and QualNPS values were analysed for potential correlations with face mask characteristics using Pearson’s correlation coefficient (r) to assess strength and significance.

#### Selection of Test Conditions

Test conditions were selected to represent field hockey impact scenarios. Three temperatures (ambient: 22 °C, cold: 5 °C, and hot: 40 °C) were taken from the field hockey ball standard to reflect various playing climates [24]. Masks and balls were conditioned at these temperatures for 4–24 hours before testing, following cricket PPE and field hockey ball standards [24, 27].

Masks were tested at nominal 60 mph (26.8 ms^-1^ ± 0.25 ms^-1^; mean ± SD), per the NOCSAE field hockey goalkeeper helmet standard [26], and 80 mph (35.8 ms^-1^ ± 0.25 ms^-1^; mean ± SD), representing the higher speeds of hits and drag flicks in elite field hockey [18, 19].

**Fig 2:**
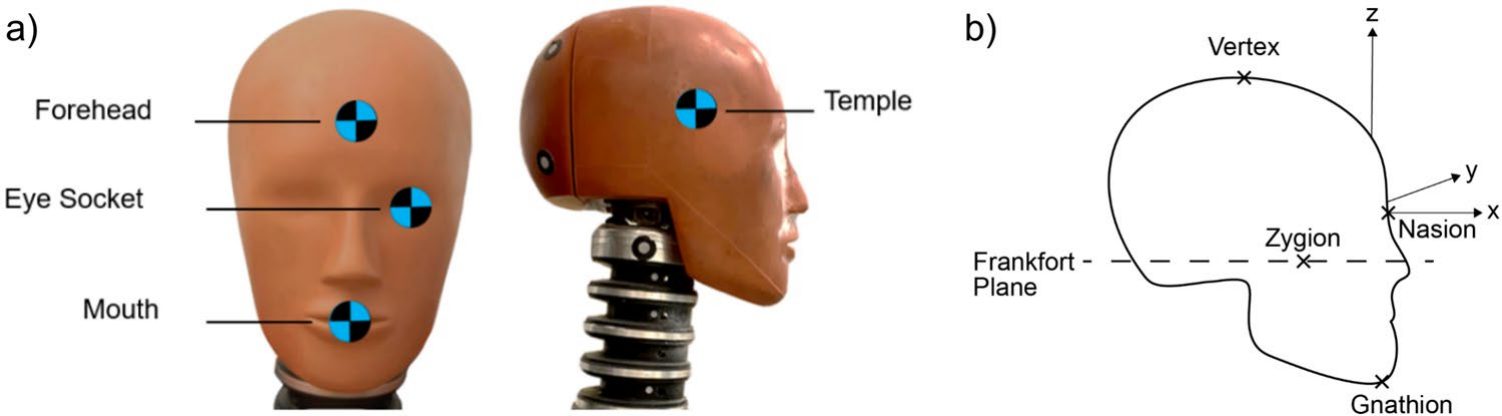
**a** Four impact locations on the Hybrid III headform. **b** Landmark locations and coordinate system. Adapted from [54]

Four anatomical locations often associated with the highest risk were selected for impact (Fig 2a).

i. *Forehead:* Due to the risk of brain injuries (such as haematoma, contusions, lacerations, and concussion) and fractures of the skull and face [48–50].
ii. *Temple:* Due to the vulnerability of the underlying meningeal artery [51] and risk of epidural haematoma leading to fatality in the worst case. Additionally, players may exhibit an anticipatory response to the approach of a ball and turn away to protect the face, putting the temple into the ball flight path.
iii. *Eye socket:* Due to the risk of serious, life changing injury; including permanent loss of vision [52].
iv. *Mouth:* Due to the risk of jaw fractures, dental injuries, and lip lacerations [53].

These four impact locations were translated onto the Hybrid III using the landmark locations, coordinate system and external geometry defined by the Hybrid III specifications for the 50^th^ percentile male headform. External landmarks and the coordinate system of the Hybrid III are shown in Fig 2b. Impact locations were defined by coordinates relative to the nasion (Table 3), where the nasion position was defined by the HIII specifications for the 50^th^ percentile male headform [54].

**Table 3:** Definition of impact locations

		Coordinates Relative to the Nasion in the Impact Plane (mm)		
Anatomical Definition	Geometric Description	x-axis	y-axis	z-axis
Forehead	Mid-way between the Nasion and Vertex	0	0	45.5
Left Eye Socket	50% between the Origin and the Frankfort Plane in line with the Zygion.	0	27	− 17
Mouth	Central and inferior from the Origin towards the Gnathion	0	0	− 79
Right Temple	Headform must be rotated 90° anti-clockwise around the z axis	− 52.5	0	0

The testing order involved each mask receiving two impacts: Either right temple then left eye socket, or forehead then mouth. The first impact (temple or forehead) was the one considered to be arguably more severe than the second. Due to the potentially destructive nature of tests and limited mask availability/cost constraints, repeat testing was not possible. Therefore, to conduct testing at four impact locations, two test speeds, and three temperature conditions, twelve samples were required per mask design. The bare headform was also impacted for comparison.

## Results

### Qualitative Testing

Qualitative results for fit, coverage, headform contact and mechanical failure are given in Table 4

**Table 4:**
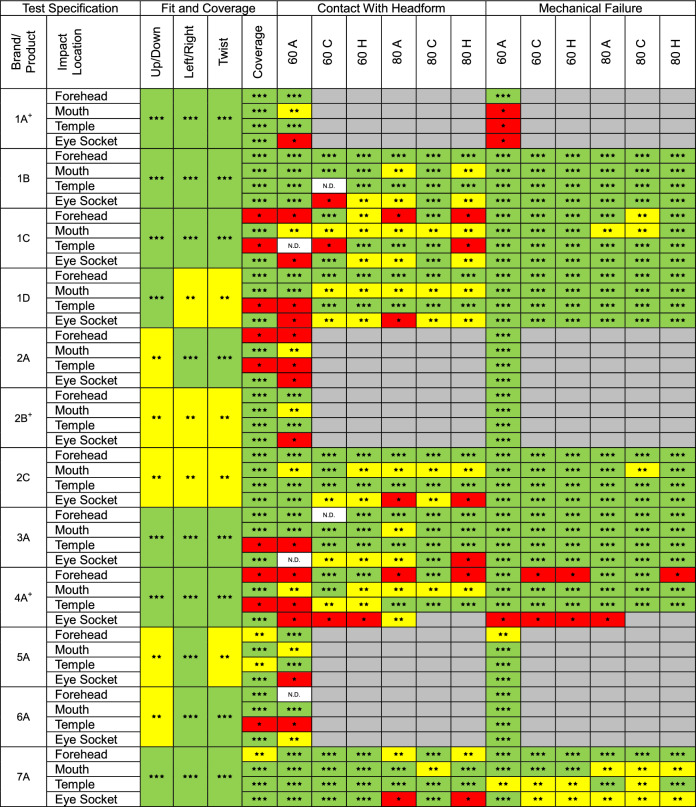
Coded qualitative grading results for fit, coverage, headform contact and mechanical failure for masks subjected to the full test battery

In all categories scoring is used as follows: (⋆) area of concern, (⋆⋆) some concern, (⋆⋆⋆) no concern.

Levels of concern by category:

Fit rating: (⋆) very loose, (⋆⋆) moderate movement under light manual manipulation in direction stated, (⋆⋆⋆) no movement under light manual manipulation in direction stated.

Coverage: (⋆) target not covered by a solid layer or within 10 mm of the edge of a foam only region, (⋆⋆) target covered by a foam only region more than 10 mm from the edge or within 10 mm of the edge of a solid layer, (⋆⋆⋆) target covered by solid layer more than 10 mm from the edge.

Contact with headform: (⋆) ball made direct contact with the headform, (⋆⋆) ball forced the hard layer of the mask to contact the headform or ball impacted a foam only region, (⋆⋆⋆) no clear contact.

Mechanical failure: (⋆) severe fracture, (⋆⋆) minor damage, (⋆⋆⋆) no visible damage.

Where no score is given, the mask had been removed from testing.

+ No longer sold by the brand/manufacturer, legacy stock still on sale.

N.D. No video data.

#### Fit

No mask received the lowest possible constraint score in any direction, indicating no instances of exceptionally poor fit with the headform. Six masks (1D, 2A, 2B, 2C, 5A and 6A) exhibited moderate movement in one or more direction; the remaining six masks (1A, 1B, 1C, 3A, 4A and 7A) exhibited little to no movement in any direction.

#### Coverage

All masks effectively covered the mouth and eye socket in the static coverage test; however, eight of twelve were insufficient at the forehead and/or temple. At the forehead, the lowest score (⋆) was awarded to masks 1C, 2A, and 4A. 5A and 7A received a score of ⋆⋆. At the temple, the lowest score was awarded to masks 1C, 1D, 2A, 3A, 4A, and 6A. 5A received a score of ⋆⋆.

#### Contact with the Headform

At least once instance of direct contact occurred at the forehead (1C, 2A and 4A), temple (1C, 1D, 2A, 3A, 4A and 6A) and eye socket (all masks except 6A). At least one instance of mask-headform contact occurred at the forehead (1C and 7A), mouth (all masks except 6A), temple (1C, 1D and 4A), and eye socket (1B, 1C, 1D, 2C, 3A, 4A and 6A).

#### Mechanical Failure

Seven masks suffered permanent damaged to varying degrees of severity. Multiple instances of severe failure occurred for 1A and 4A, and minor damage occurred for 1B, 1C, 2C, 5A and 7A.

### Impact Testing

**Fig 3:**
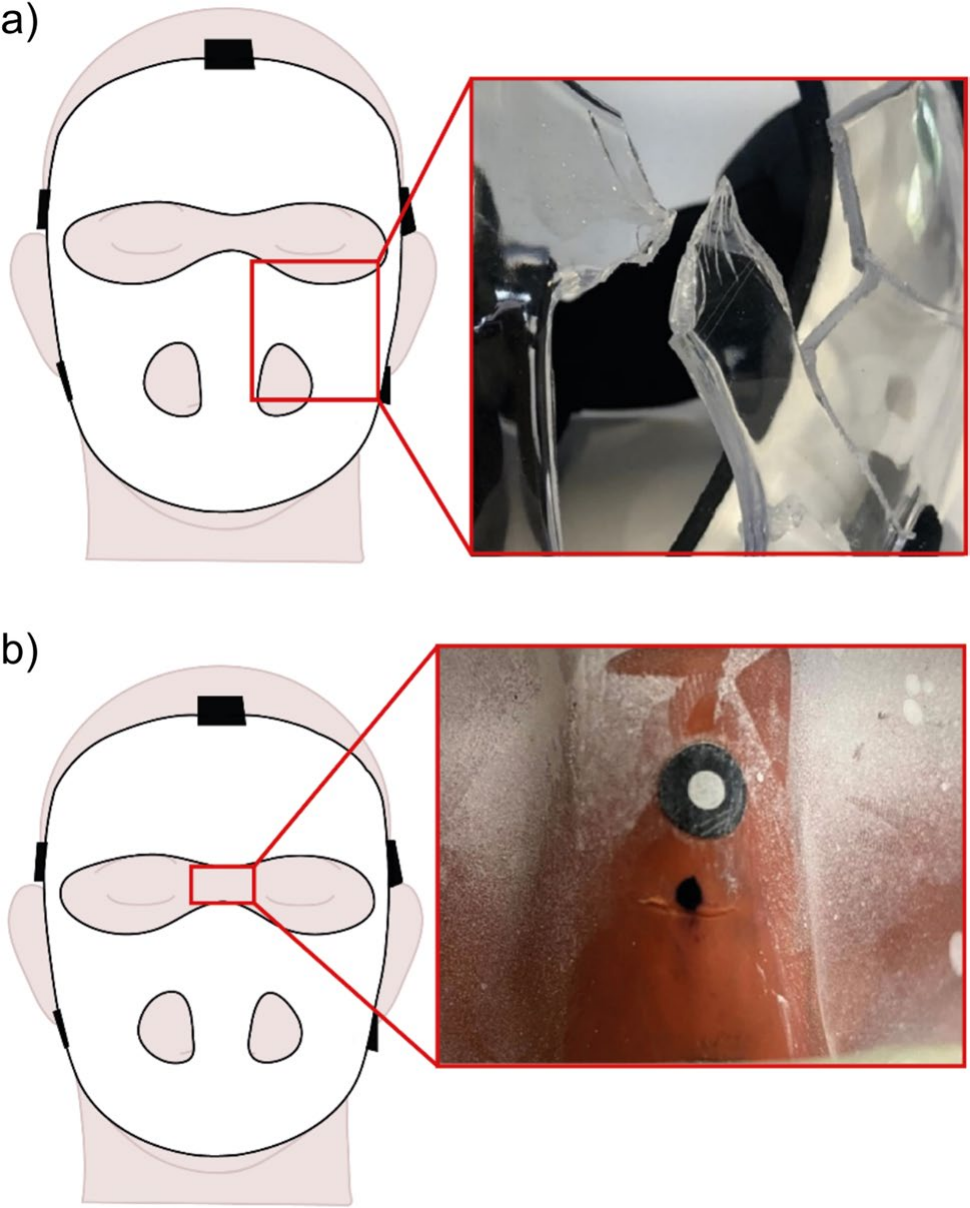
**a** An example of mechanical failure. Significant material fracture with fragments detached from mask leaving sharp point behind. **b** An example of damage to the nose of the HIII headform caused by a face mask

All twelve masks were subjected to 60 mph impacts at ambient temperature. Five masks were excluded from further testing: Two (1A and 2B) were no longer commercially available, and three (2A, 5A and 6A) caused damage (or risk of damage) to the headform’s vinyl skin. The individual performance of these masks was generally amongst the least protective. Examples of damage to a mask and to the headform are given in Fig 3a, b respectively. Performance of such masks raised safety concerns that were immediately drawn to the attention of the FIH.

**Fig 4:**
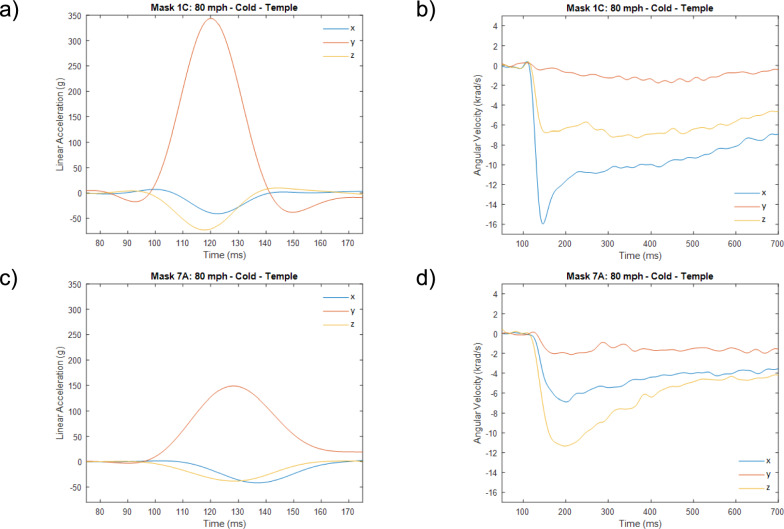
Sample filtered responses for masks at both ends of the performance spectrum when impacted at 80 mph under cold conditions at the temple location. **a** Linear acceleration traces for mask 1C. **b** Angular velocity traces for mask 1C. **c** Linear acceleration traces for mask 7A. **d** Angular velocity traces for masks 7A

Exemplar linear acceleration and angular velocity response traces are provided in Fig 4a–d for examples of masks at either end of the performance spectrum.

In the following sections, results are presented for the seven masks tested under all conditions. Results are presented in bar chart form, where each solid-coloured bar represents a different temperature condition at 60 mph (green-ambient, red-hot, blue-cold); the corresponding 80 mph impact results at the same temperature are represented by the white bar with black border extension to the coloured bar of the appropriate temperature/colour. The bare headform results are displayed as dotted and solid lines for 60 mph and 80 mph impacts respectively, to allow for comparison between bare headform and masked conditions.

#### Resultant Peak Linear Acceleration

**Fig 5:**
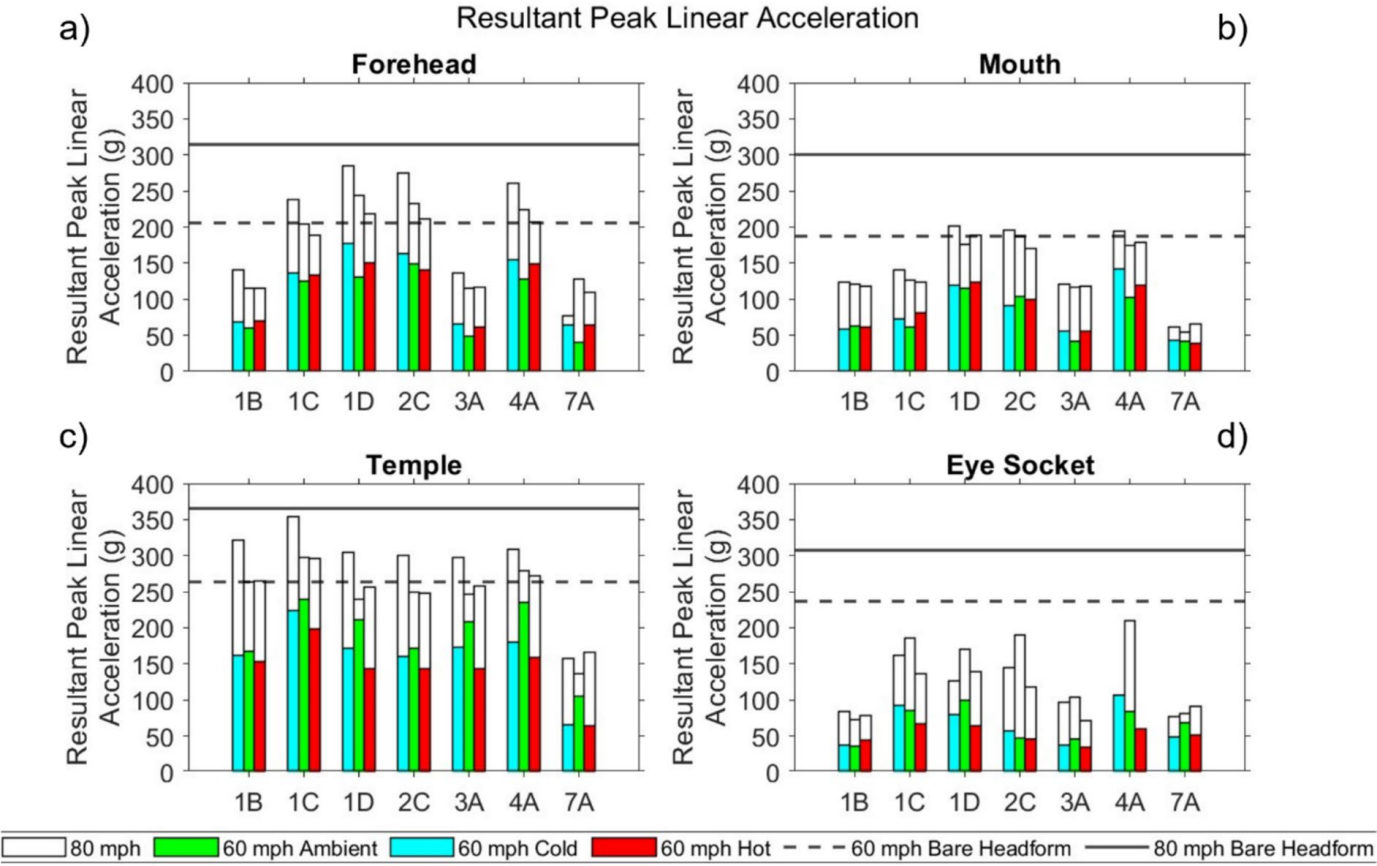
Resultant PLA for masks tested at all three temperatures at the **a** forehead **b** mouth **c** temple **d** eye socket

Resultant peak linear acceleration (PLA) results from the full test battery are given in Fig 5. For reference, a threshold of 250 g is the accepted threshold used in British Standards for cricket and cycling [27, 46] and is reported as corresponding to an estimated 40% risk of skull fracture [41].

When impacting the bare headform, the temple was the only region exceeding the 250 g threshold at 60 mph; all locations exceeded the threshold at 80 mph for the bare headform.

When impacting the masked headform at 60 mph, all masks lowered PLA below the 250 g threshold at all locations and temperatures. However, at 80 mph, the threshold was exceeded at the forehead and temple, highlighting varying degrees of performance between masks. Compared to other impact locations, the temple had consistently high PLA values across test conditions and masks models. Of the masks subjected to the full test battery, only 7A was able to reduce PLA below the bare headform value at all temperatures, locations, and speeds.

#### Head Injury Criterion

**Fig 6:**
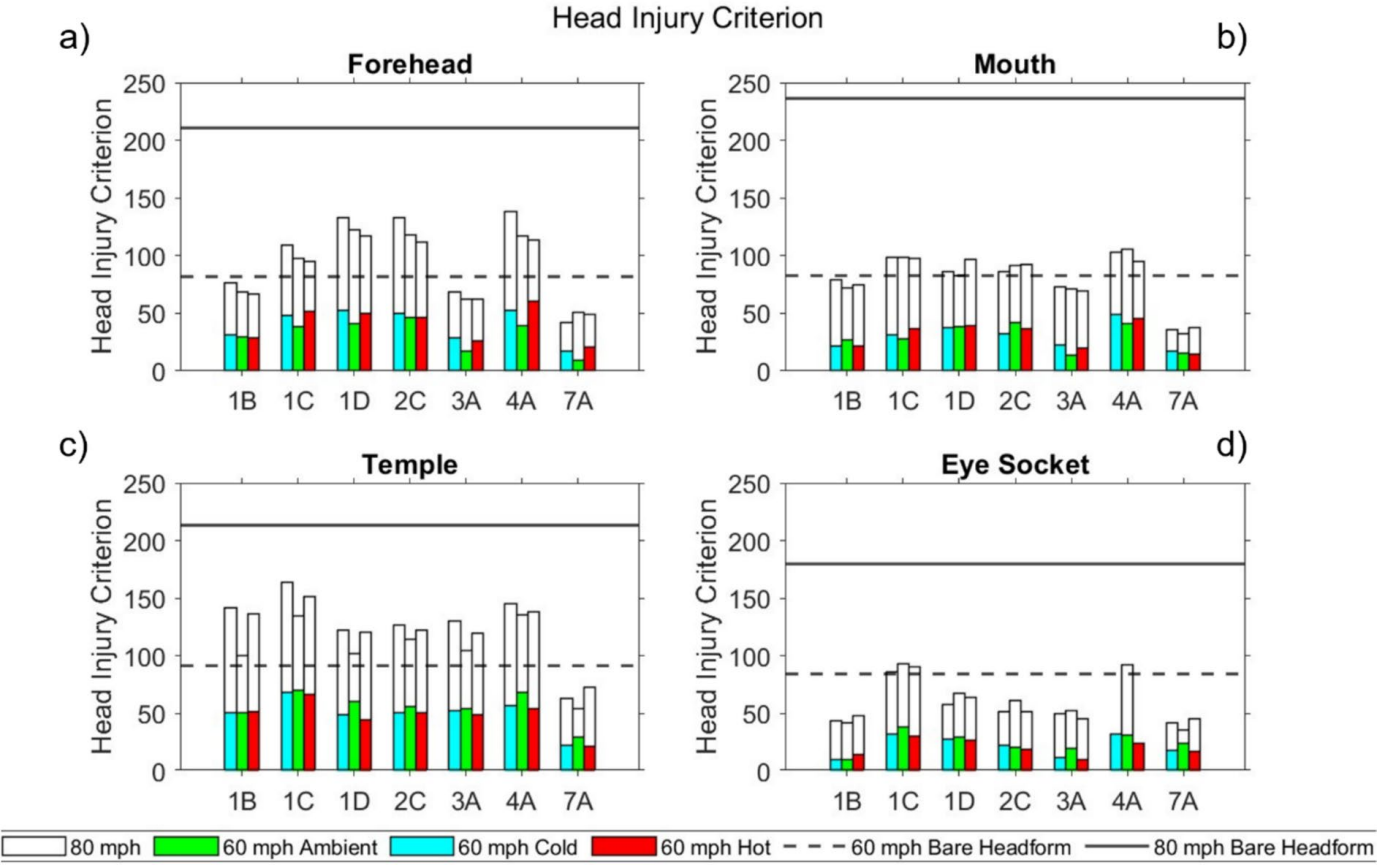
HIC for masks tested at all three temperatures at the **a** forehead **b** mouth **c** temple **d** eye socket

Head Injury Criterion (HIC) results are presented for masks subjected to the full test battery in Fig 6. For reference, the US airbag test standard [47] states an injury threshold of 700 which corresponds to an estimated 5% risk of severe (AIS 4 + ) injury [41].

For the bare headform, the HIC score was significantly lower than the published injury threshold of 700 [47] at all locations, temperatures, and impact speeds.

All masked impacts were below both the published injury threshold of 700 and the bare headform value at all impact conditions. Consistent with PLA results, HIC values at the temple region were high across test conditions and masks. 7A was the best performing mask across all test conditions, and was the only mask able to consistently reduce impact levels at 80 mph to below those experienced by a bare headform at 60 mph.

#### Resultant Peak Angular Acceleration

**Fig 7:**
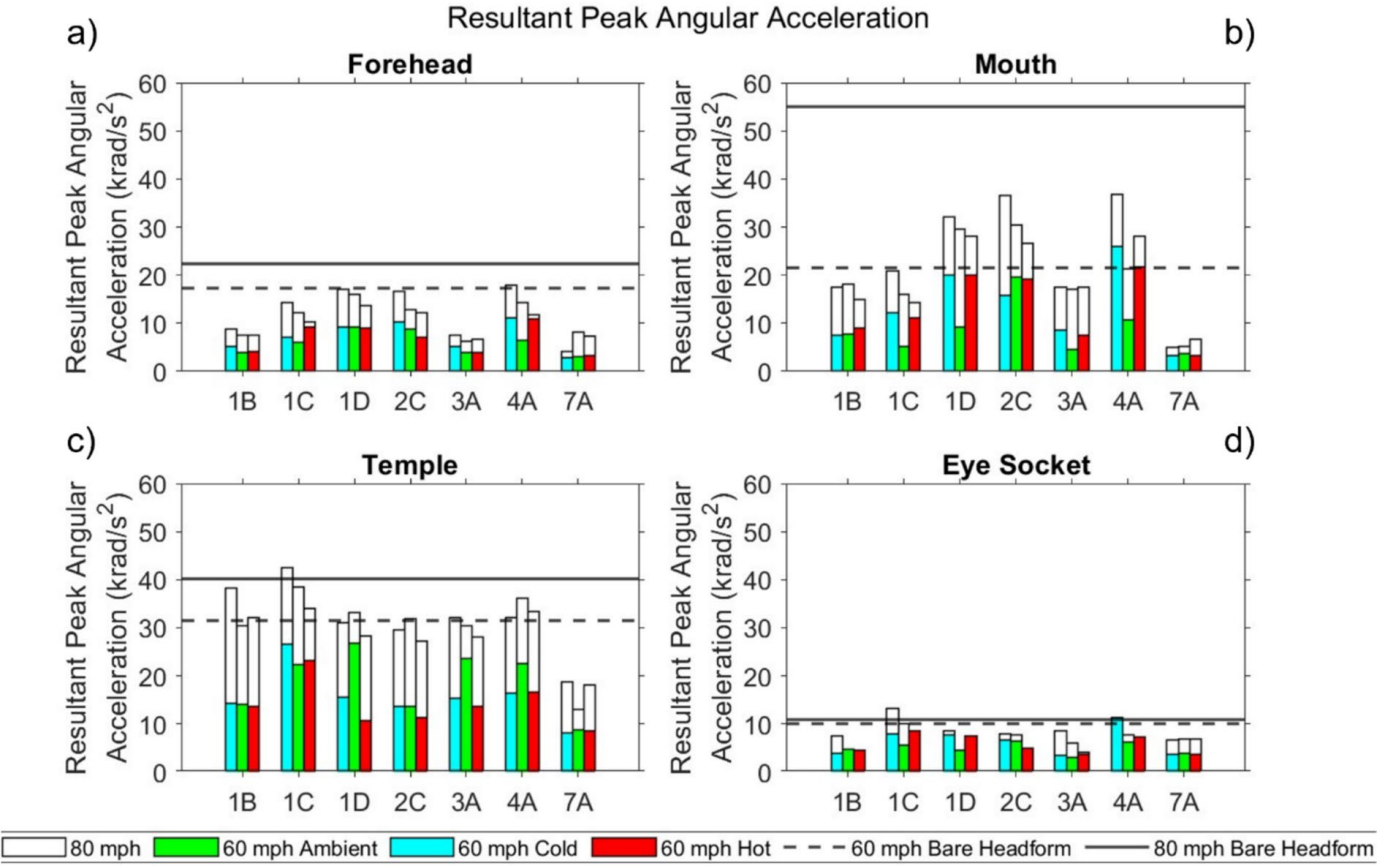
Resultant PAA for masks tested at all three temperatures at the **a** forehead **b** mouth **c** temple **d** eye socket

Resultant peak angular acceleration (PAA) results are presented for masks subjected to the full test battery in Fig 7. PAA is not widely used in test standards; for reference, Funk et al. [43] propose 9,000 rad/s^2^ which corresponds to a 10% risk of mTBI in American Football applications.

For the bare headform impacts, PAA was above the published injury threshold of 9,000 rad/s^2^ [43] at all locations, and speeds.

For masked headform impacts, in several instances PAA exceeded the published threshold at all impact locations, most notably at the temple, where all masks exceeded the threshold at all impact temperatures and speeds (except 7A when tested at 60 mph).

At 60 mph, all masks lowered PAA compared to the bare headform condition at the forehead and temple at all temperatures. However, at the mouth and eye socket there were instances where masks exceeded the bare headform condition. At 80 mph, all masks lowered PAA compared to the bare headform condition at the forehead and mouth at all temperatures. At the temple and eye socket, the bare headform value was only exceeded at the cold temperature by 1C. Consistent with PLA and HIC score, the temple also had some of the highest PAA values across conditions.

#### Brain Injury Criterion

**Fig 8:**
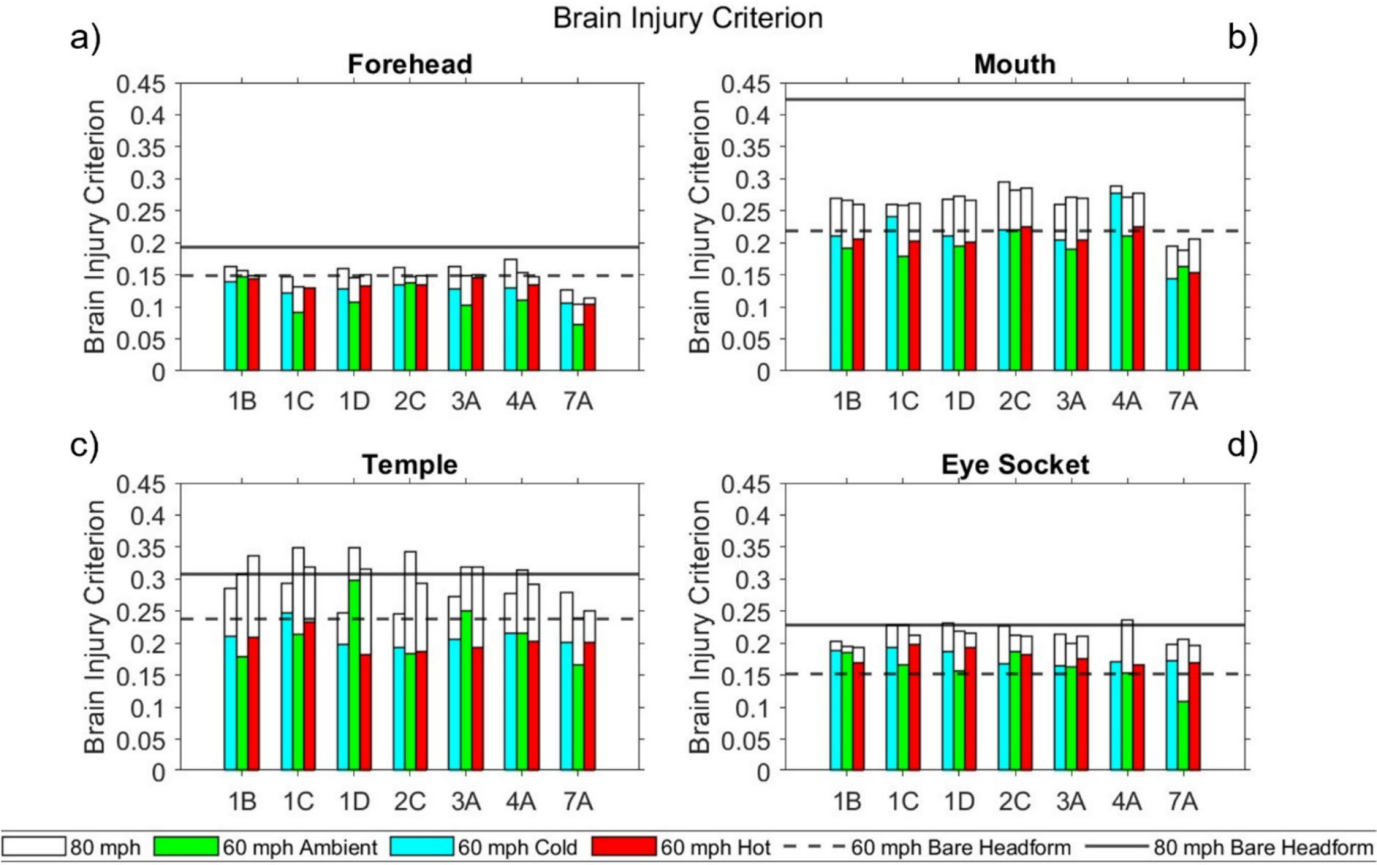
BrIC for masks tested at all three temperatures at the **a** forehead **b** mouth **c** temple **d** eye socket

Brain Injury Criterion (BrIC) results are presented for masks subjected to the full test battery in Fig 8. BrIC is not used in test standards. For reference, Takhounts et al. proposed that a BrIC score of 1.0 corresponds to an estimated 30% risk of an AIS 4 + injury [39] in an American Football context.

For the bare headform impacts the BrIC score was significantly lower than the threshold of 1.0 [39]. Similarly, all masked headform BrIC scores were less than 1.0.

At 60 mph, all masks lowered the BrIC score compared to the bare headform condition at the forehead at all temperatures. However, there were instances where the BrIC score exceeded the bare headform value at the mouth, temple, and eye socket. Notably, at the eye socket, this occurred for all masks at all three temperatures, except 7A at ambient temperature. At 80 mph, all masks lowered the BrIC score compared to the bare headform condition at the forehead and mouth at all temperatures. Consistent with the other three metrics, BrIC scores at the temple were high across test conditions and masks.

Unlike for the other three metrics calculated in this study, no mask reduced the BrIC score below bare headform values for all test conditions, although some were closer than others.

#### Net Performance Scores

There were no correlations found between QualNPS and any mask characteristic. A moderate positive correlation was found between QuantNPS and hard layer thickness (*r* = 0.66, *p* = 0.020) and QuantNPS and total foam layer thickness (*r* = 0.69, *p* = 0.014). Both correlations were statistically significant (*p* < 0.05), indicating that increasing hard or foam layer thickness is generally associated with improved quantitative mask performance, where coverage extends to the impact location concerned. Specific foam type (e.g., open or closed cell), shell layer and foam materials, and primary and secondary forming processes (e.g., compression moulding of pre-blown foams that alter foam density) might also be expected to influence performance and most likely account for the moderate correlation with thickness. More extreme variation in fit might also be expected to have a stronger influence on protective outcome, but this is not proven here.

## Discussion

### Methodology

The research described has successfully used quantitative and qualitative test methods, informed by other sports’ standards and research testing, to assess a range of commercially available field hockey face masks. The results provide a previously unpublished benchmark for impact performance, highlighting safety concerns, and identifying the need for better field hockey-specific test equipment and methods to more adequately explore facial impact severity in the future and subsequently improve safety within the sport.

#### Samples and Repeat Impacts

The range of masks tested represented designs available across the concept-sparse outfield hockey face mask space. It has been demonstrated that masks apparently of the same type (i.e., plastic shell construction) will not necessarily deliver the same protective performance—a previously unproven and useful insight for the field hockey player community that highlights the need for consistent test standards and the publication of results for the industry to reassure its customer base. Brands are not identified in this publication as a condition of participation in the study, but it is hoped that anonymous publication of the wide range of performance discovered will encourage brands to be more candid about their successes, and responsible with their failures, in future.

In the future, each size of the various designs might also be tested, as is standard for other sports’ headgear, but, as a first foray into hockey outfield mask testing, use of only the more commonly available 50^th^ percentile male anthropomorphic test device (ATD) components has provided useful insights.

Repeat impacts at the same impact site might provide additional insights into average and worst case performance (requiring more new masks to be tested); similarly, mask durability might be studied (requiring the same masks be hit more times). Nonetheless, the approach utilised has kept the research cost-effective and reduced the initial burden on collaborators. That said, six masks of each design have been subject to multiple impacts at the same nominal locations (albeit at different temperatures) increasing the likelihood that manufacturing process variability, resulting in different performance outcomes at the same location for masks of the same design, would become apparent if problematic.

#### Balls and Temperatures

The use of real field hockey balls conditioned, with the masks, at temperatures accepted by the industry as relevant to field hockey equipment performance adds validity to the study, but also some cost. The addition of both cold and hot temperatures to ambient testing is useful to expose the potential for catastrophic brittle failure or excessive deformation (and so facial contact). The variability in design performance amongst the masks tested demonstrates that performance at different temperatures is not entirely predictable, for a given design type, necessitating testing at a range of use temperatures to adequately ensure safety.

#### Test Rig, Speeds, Precision and Impact Locations

The iBPR rig used provided reliable speed and precision enabling fair testing with a relatively low number of sample masks, i.e., without necessitating repeat impacts with ‘additional/spare’ masks due to frequent off-target impacts or nominal speed fluctuation.

The relevance of the speeds used to the game of field hockey has added validity and context to the results, providing insights for elite and recreational players alike. The range of precisely targeted impact locations chosen, and the justification for them, have revealed the limitations of some design approaches when protecting critical areas of the head (i.e., the temple).

#### Measures and Instrumentation

The combination of the HIII headform, neck and 6DX Pro instrumentation performed as expected to monitor whole-head kinematics, in response to impact, and enabled consideration of a range of published brain injury criteria. Some indication of impact force necessary to cause this response is implied by the results, but the local magnitude and distribution of pressure on the face during impact remains indeterminate.

Whilst providing a useful visual reference for the impact event, the use of HSV proved inadequate, as perhaps expected, to objectively determine the local severity of ball-mask-face interaction. Contact spray was not used in anticipation of similar limitations: Contact can be seen but not quantified. Alternative headforms are commercially available to measure the local contact force, but at a cost beyond the scope of this research. The development of more cost-effective solutions could usefully be pursued in further work.

### Findings—Qualitative Testing

#### Mask Fit

No mask received the lowest fit score in any direction, indicating no cases of exceptionally poor fit with the headform, but it should be noted the HIII headform skin is regarded as having a higher coefficient of friction than human [55], this could affect the way masks behave under application of light manual manipulation compared to how they might behave when worn by players.

Constraint systems on some masks could have been affected by the lack of biofidelity in the basilar region of the HIII. If the strap cannot sit on the headform as intended, the mask could be less well constrained than intended and therefore have a poorer impact response. However, this was mitigated by ensuring a reasonable and repeatable fit with the headform, despite the slight deviations from manufacturer’s fitting instructions, limiting potential effects on impact response from poor fit. Additionally, this did not become apparent in the outcomes of fit or impact testing; 1A appeared to be the worst fitting in this region but was not an outlier for any metric presented.

Given field hockey masks are usually available to purchase in unisex senior and junior sizes, investigation into whether such sizes provide sufficient fit for both male and female players could be important, especially due to the 50:50 gender split in field hockey globally.

Assessments of fit and constraint are influenced by the head surrogate used, therefore, to investigate the effect of biological sex on mask fit the size and geometry of the headform used requires further consideration. However, as a measure of test fairness in this study, none of the differences in performance outcomes observed appear to be attributable to poor fit in combination with the chosen headform.

#### Coverage and Contact with the Headform

Only six of twelve masks had sufficient coverage (⋆⋆ or ⋆⋆⋆) at all four locations.

In particular, at the temple; seven out of twelve masks received a score of ⋆ or ⋆⋆. Due to the potential for severe or lethal injury at this location, it is concerning that 58% of masks tested did not score more highly, particularly given that players may exhibit an anticipatory response to the approach of a ball, e.g., turning away from an impact to protect the face, but potentially placing the temple in the balls path. All masks experiencing direct contact at the temple were those deemed to have partial or no coverage in that location. However, 5A received a score of ⋆⋆ and did not have any instances of observed direct contact, despite providing less coverage than the rest of the mask cohort. This is possibly due to modest impact location variation possible with the iBPR (± 11.3 mm) or the limitations of contact observation.

All masks effectively covered the mouth and eye socket in the static coverage test. Additionally, there were no instances of direct contact at the mouth, indicating the mouth opening of all masks was adequate to prevent gap penetration. There were instances of mask-headform contact, but the severity of these interactions cannot be determined from HSV. There is the potential for this type of interaction to cause injury to the skin, particularly if the edges of the opening are sharp. Despite the sufficient eye socket coverage for all masks, every mask exhibited at least one direct contact, suggesting masks are ineffective at preventing gap penetration at this location. In the cricket helmet standard [27], the grille penetration assessment uses a more rigid headform mounting as a neck alternative to provide maximum inducement for the ball to penetrate the grille-visor gap. This study’s use of the acknowledged overly stiff HIII neck may have had a similar, though reduced, effect (given the similarly brief impact event time, 4–6 ms). Nonetheless, comparison of results with respect to facial contact when using a more biofidelic neck could and is intended to be the subject of future work.

Whilst instances of direct contact were identified, the test conducted (i.e., review of HSV) is open to perspective errors and cannot provide an indication of impact duration or severity (e.g., peak contact force or pressure distribution). In most examples contact or non-contact was obvious; but, on some occasions categorisation was challenging.

In some test standards (e.g., the NOCSAE standards for head protection in ice hockey, baseball/softball, lacrosse and field hockey goalkeepers), ‘pressure indicator’ paste is used as a contact witness, as is contract witness spray in the British Standard for cricket helmets [26, 27, 56–58]. Compared to HSV this approach requires more effort and yields little additional insight. In future field hockey face mask testing, the introduction of an instrumented face region to give an objective measure of contact severity is desirable, if cost-effective.

#### Mechanical Failure

Table 4 indicates that several masks exhibit mechanical failure under hot or cold test conditions that is not seen at ambient temperatures for both speeds (i.e., at 60 mph: 4A forehead and 7A eye socket at both hot and cold temperatures; at 80 mph: 1C forehead, 2C mouth, and 7A temple at cold temperature, and 4A forehead at hot temperature). This implies temperature as well as impact speed might be a significant factor in mechanical failure at these locations for some masks, although this might also be an issue with the individual mask, or variation in impact location within the equipment repeatability. Therefore, to cover all eventualities, future testing should include hot, cold, and ambient temperature testing to identify weaknesses not exposed or predicted from ambient temperature testing alone.

No mask experiencing these failures specified in their online literature the polymer used, but there were other polymer masks that did not experience mechanical failures. None of the damaged masks were significantly thinner than others in the study (5A was thinner than average but did not experience severe damage). Material, thickness, shape, and manufacturing process can all contribute to mechanical failure; therefore, it is not possible to legitimately rule out masks based on material alone to avoid the cost of testing.

Interestingly, the metal grille type mask sustained more minor, permanent damage than the plastic type, due to the material exceeding its yield point. Despite these instances, the metal mask had less instances of both direct ball-headform and ball-mask-headform contact. Whilst the metal mask did not suffer any severe mechanical damage, it is theoretically possible for alternate metal wire designs to fail (particularly where materials are joined). This provides the potential for metal pieces to protrude towards the face, posing a risk of ocular injury; thus, even metal construction masks require testing to prove their adequacy (as in the case of the mask tested).

### Findings—Quantitative Testing

#### Peak Linear Acceleration and Head Injury Criterion

All masks subjected to the full test battery reduced PLA compared to the bare headform for all impact conditions; the same was true of HIC. The published reference injury thresholds were not exceeded for either metric, except PLA for some instances of 80 mph testing at the forehead and temple.

HIC was developed based on Gadd Severity Index research, which included both long and short duration impacts, and is often used in the context of automotive crashes. Although automotive impacts are typically of longer duration than those in field hockey, HIC15 (the version used in this study) was designed to accommodate impacts of shorter duration, such as those observed here (ball-face mask contact time was estimated from HSV to be in the region of 4–6 ms). The HIC15 metric is valid for evaluating impacts of this duration, but the reference threshold of 700—selected from an existing published automotive standard—may be suboptimal for field hockey, given the range of thresholds reported in the literature for achieving different levels of protection (Table 2), it is perhaps at best a guide in the absence of field hockey-specific injury data, and industry consensus on what levels of protection should be expected.

Additionally, the selected 250 g reference threshold for PLA (taken from drop testing scenarios with longer contact time) is also at least partially mismatched to the risk of projectile impacts in field hockey.

Future work could aim to improve the appropriateness of PLA and HIC metrics and thresholds for use in a potential field hockey headgear test regulation. In the absence of optimal thresholds, a comparison of the masked outcomes with unmasked impact results has provided a useful contextual and meaningful reference for the industry, familiar with the real-life outcomes of unmasked ball-face impacts.

#### Peak Angular Acceleration and Brain Injury Criterion

Unlike PLA and HIC, PAA is not widely used in test standards. The NOCSAE standard for American Football helmets uses 6,000 rad/s^2^ as a threshold in pneumatic ram tests [59]. However, these impacts can have a slightly longer impact duration (8.99 ± 3.01 ms [60]) than the 4–6 ms field hockey impacts observed in this study. The 10% risk of mTBI at 9,000 rad/s^2^ reference threshold by Funk et al. [43] was accompanied by equivalent risk thresholds of 165 g (PLA) and 400 HIC for the linear metrics reported; this corresponds to decreases of 34% and 43% respectively compared to the more widely used thresholds. This disagreement in another sports context with more commonly accepted thresholds from other contexts perhaps indicates that Funk et al.’s proposals are conservative, but it more certainly indicates the need for caution when referencing thresholds in a different context.

Masked headform BrIC scores were significantly lower than the published reference threshold for the bare headform and all masks tested at all impact conditions. Unlike the other metrics used in this study, no mask reduced BrIC below both the reference threshold of 1.0 and the bare headform value for all test conditions. There was evidence that wearing a mask can increase BrIC score, especially at the eye socket, possibly due to the presence of a mask providing the ball a longer moment arm about the centre of mass for the impact force, and the geometry of the eye opening providing a ‘pocket’ in which the ball finds greater mechanical coupling with the mask-headform system.

Based on the other metrics, the eye socket appears to have the lowest risk; but BrIC scores show it is also an area of concern for mTBI. This, in combination with the demonstrated potential for facial contact and severe mechanical failure at this location, highlights the eye socket as an area of concern that cannot be addressed using PLA or HIC alone. Thus, future work could usefully consider measurements, in this zone, to fully encompass facial injury risk.

Thresholds optimised for field hockey have yet to be established for each of the injury metrics calculated in this study. It is the responsibility of the industry and governing bodies to determine acceptable injury risk levels for the sport. This will help ensure that consumers are informed about the minimum performance they should expect of the masks they are purchasing.

#### Net Performance Scores

It is known that specific material properties, shape, and thickness influence the effectiveness of PPE at a particular impact site; attributing performance outcomes to a sole design characteristic would generally be an oversimplification.

However, examining several designs on the market currently (i.e., immediately prior to publication)—each representing combinations of these factors—revealed two notable trends in the quantitative assessment of performance. A moderate positive correlation was found between QuantNPS and both hard layer thickness, and total foam layer thickness. This suggests that increased thickness in these layers generally contributes to improved quantitative performance.

In contrast, no significant correlations were observed between QualNPS and any face mask characteristic. This suggests that favourable qualitative performance may result more from design serendipity than the physical properties measured here.

#### Limitations

Finally, it should be noted that the differences in mask performance trends between the linear (PLA and HIC) and angular (PAA and BrIC) metrics could be contributed in part by the ATD used. The HIII neck was developed primarily for frontal impacts, meaning it responds with greater biofidelity (although overly rigid) in the sagittal plane (rotation about the y-axis, cf. Fig 2b), the dominant axis of rotation in the forehead, eye socket and mouth impacts. Temple impacts had a dominant axis of rotation in either lateral flexion or axial rotation (x or z), depending on exact impact location and mask shape. The HIII neck has even less biofidelity (excessive rotational rigidity) in these axes [61]. By using the HIII neck, known to be stiffer than an unbraced human response, the early impact rate of force development is exaggerated and total contact time reduced. The excessive neck stiffness is also likely to result in lower-than-human head accelerations. Further work could usefully explore the effects of using a more biofidelic neck to improve the validating of these assessments.

There has been no research to correlate levels of PLA, HIC, PAA, or BrIC with the likelihood of injury in a field hockey context previously and is beyond the scope of this study. However, it would be unhelpful to provide impact assessment results based on these metrics without some reference to acceptable levels in other contexts. The research summarised in Table 2 does identify metric levels associated with relevant types of injury in other head impact contexts. In the absence of field hockey-specific research data, the levels quoted do provide context for the results presented here, until field hockey-specific thresholds can be established. Use of any of the previously reported levels here as a reference value is not intended to suggest that a corresponding likelihood of injury exists in a field hockey context.

Thus, there remains a need for research into the optimal metrics and thresholds for use in field hockey. However, in a context where improvements must be made on a short timescale, for the safety of players, it is prudent for this work to include reference to exemplars, until such a time as more suitable metrics and thresholds have been established for these impact scenarios.

#### Implications for the Industry

The results of this study highlight the need for a test regulation for field hockey face masks. The inclusion of multiple temperatures, speeds, and locations revealed inconsistent protective performances across the range of masks, with key points of weakness including mechanical failure, facial contact, and the routine excess of PLA above the widely accepted, but not cautious, threshold of 250 g at match realistic speeds. Broader industry alignment on these characteristics alone could considerably improve safety in field hockey at all playing levels. Although various threshold levels that might be used as a pass/fail criteria for mask performance have been presented, it remains the work of governing bodies and industry stakeholders to determine which level is appropriate for the sport informed by this research.

Following testing, results were shared with the FIH and collaborating equipment brands. This has already led to model revisions that now pass the PLA and material failure criteria at the 60 mph speed (1A revised to 1B and 2B revised to 2C) and is evidence that this research has already significantly impacted safety in field hockey. Further benefits to the sport in terms of improved face mask designs from all manufacturers is anticipated.

Beyond their relevance to field hockey, the methods employed in this study could be used as a starting point for the evaluation of potential future equipment to protect bowlers from ball to face impacts in cricket.
